# Reactions to a Hypothetical Menthol Cigarette Ban among Sexual- and Gender-Minoritized Communities: A Concept Mapping Study

**DOI:** 10.3390/ijerph20053891

**Published:** 2023-02-22

**Authors:** Ashlee N. Sawyer, Madison Combs, Viktor Clark, Eric K. Soule, Joseph G. L. Lee, Alison B. Breland

**Affiliations:** 1 Center for the Study of Tobacco Products, Department of Psychology, Virginia Commonwealth University, Richmond, VA 23220, USA; 2 Department of Health Behavior and Policy, Virginia Commonwealth University School of Medicine, Richmond, VA 23220, USA; 3 Department of Health Education and Promotion, College of Health and Human Performance, East Carolina University, Greenville, NC 27858, USA

**Keywords:** sexual minority, gender minority, LGBTQ, transgender and gender diverse (TGD), adults, menthol cigarettes, menthol ban

## Abstract

Menthol cigarette use is disproportionately higher among sexual- and gender-minoritized (SGM; 36%) individuals compared to cisgender, heterosexual (29%), individuals. The FDA has announced intentions to ban menthol in cigarettes, citing these use and health disparities as partial motivation. This study identified potential outcomes of a menthol cigarette ban among SGM individuals who smoke menthol cigarettes (N = 72). Potential outcomes were identified via concept mapping using the prompt: “If menthol in cigarettes was banned, a specific action I would take related to my tobacco use is…” Participants generated 82 response statements, sorted them, and rated them on personal relevance. Eight thematic clusters were identified: (1) Thoughtful Consideration of the Ban, (2) Negative Reactions to the Ban, (3) Positive Aspects of the Ban, (4) Strategies to Reduce Cravings, (5) Intent to Quit and Cessation Strategies, (6) Support-Seeking and Engagement in Positive Behaviors, (7) Strategies to Maintain Menthol-Flavored Product Use, and (8) Substance Use Alternatives to Menthol Cigarettes. Cluster differences based on sociodemographic factors, smoking behavior, and quitting interest were identified. Results provide insight into potential responses to a menthol cigarette ban and can contribute to public health prevention and intervention efforts, messaging campaigns, and support services for SGM people who smoke menthol cigarettes, specifically.

## 1. Introduction

Lesbian, gay, bisexual, transgender, queer/questioning, plus (LGBTQ+) communities are home to a diverse collection of identities that fall under the overarching umbrella of being a sexual and/or gender-minoritized (SGM) person. Cigarette use is disproportionately high among SGM groups relative to non-SGM people. While cigarette smoking rates among the general U.S. population have fallen, with 12.5% of adults reporting current use in 2020 [1], cigarette smoking rates among LGB individuals (20.6%) and transgender adults (35.5%) remain higher [2]. Of particular concern is menthol cigarette use, which is disproportionately higher among SGM individuals who smoke (36% of SGM individuals who smoke use menthol cigarettes) versus their cisgender, heterosexual counterparts (29% of cisgender individuals who smoke use menthol cigarettes) [3]. The disparate rates of menthol cigarette use among SGM individuals are largely due to targeted marketing that is carried out by the tobacco industry, pride-specific marketing, and the funding of pride festivals [4].

Menthol cigarette flavoring enhances the taste of smoke, decreases the unpleasantness associated with early smoking, and creates a significant barrier for adults attempting to quit smoking. However, previous literature has also indicated that menthol smokers are as motivated or more motivated to quit smoking than non-menthol users. Prior work has also reported that more than half of all Americans are supportive of banning menthol cigarette use and would support an FDA action to ban menthol cigarettes [5]; however, these rates have not been stratified by LGBTQ+ identity. This is a current gap, as LGBTQ+ communities are high-risk communities for cigarette use [1]. As SGM individuals are more likely to smoke menthol cigarettes and menthol cigarette use is associated with higher rates of nicotine dependence and poor cessation outcomes [6,7], cigarette use and resulting morbidity and mortality in this population are of particular concern. Importantly, menthol cigarette use is also high among other groups, such as Black individuals (e.g., [7]), and much work has been conducted with Black individuals who smoke cigarettes (for review, see [7]), but much less work has focused on SGM people who smoke menthol cigarettes.

Many health agencies, including the American Lung Association (ALA), the Centers for Disease Control and Prevention (CDC), the Food and Drug Administration (FDA), and the National Institutes of Health (NIH)/National Institute of Minority Health and Health Disparities (NIMHD) have recognized the substantial tobacco use disparities that exist within the SGM community and, as a result, have named this community as a priority population for research and prevention efforts [4,8,9,10,11,12]. As a result, the research on smoking prevalence and tobacco use behaviors within this community has grown, although more work is needed on attitudes and behaviors (as noted in [13]). The increased availability of data highlighting health disparities and the role of systemic and societal factors has led to the development of public health initiatives, including tobacco regulatory efforts. Research is mixed on attitudes toward tobacco regulation; some shows that people who smoke cigarettes view tobacco regulation of some products positively (e.g., [14]), and in a small sample of community leaders working in the LGBTQ+ community, support for tobacco regulation was strong [15]. However other research (with an LGBTQ sample) showed low support for some tobacco control policies [16].

The FDA has announced its intention to ban menthol in cigarettes, citing SGM individuals’ disparate use of menthol cigarettes and the goal of reducing health disparities within this group as part of the motivation for pursuing the ban [17]. Research exploring responses to a menthol ban among people who smoke cigarettes, with samples not specifically centered on SGM individuals, shows mixed responses. For example, some positive responses include quitting smoking, which could improve health outcomes, and some negative responses include switching to nonflavored cigarettes and not quitting, thus perpetuating poor health outcomes [18,19]. Although various health and community organizations have suggested that a menthol ban would improve public health outcomes within SGM communities [4,20], research has not yet been conducted to evaluate the potential responses to such a ban among SGM individuals who currently smoke menthol cigarettes. 

In order to maximize the policy’s health-promoting intentions while preventing unintended, health-harming consequences, it is imperative that policy efforts be informed by voices from the communities that are most affected by menthol cigarettes. Exploring the nuances of responses to a menthol ban across various groups would help determine how to best implement this policy change and how to create appropriate messaging. The purpose of this study was to determine possible behavioral responses to a ban on menthol in cigarettes among SGM individuals who smoke menthol cigarettes.

## 2. Materials and Methods

This study used concept mapping [21]—an integrative, mixed-methods approach—to create a model organizing and describing community-generated content regarding potential behavioral responses to a hypothetical menthol cigarette ban among SGM people who smoke menthol cigarettes. This process includes statement generation through participant completion of brainstorming, sorting, and rating tasks to create an interpretable concept “map” of participants’ responses. Details of concept mapping methods and procedures have been described previously [22], and all data collection was conducted online. Of note, there is no typical sample size for concept mapping studies. The analysis of these data allows for the examination of community-generated responses to policy change, common themes, and group differences. This methodology has been used to identify and describe behavioral responses to an implemented menthol cigarette ban in Canada [23,24] and a hypothetical e-cigarette flavor ban in the U.S. [25], among other topics. This study was approved by Virginia Commonwealth University’s Institutional Review Board.

### 2.1. Participant Recruitment and Eligibility

From November 2021 to April 2022, SGM adults who smoke menthol cigarettes were recruited through advertisements on location-specific Craigslist boards. Craigslist has previously been identified as an online space used by racially diverse SGM communities [26,27], SGM individuals living in rural areas [28], and minoritized groups more generally [29]. As diverse SGM samples are regarded as “hidden” and remain hard to access [26], SGM individuals frequently use online platforms to access community [30], and Craigslist has been reported to be an effective recruitment tool [28,31] that produces quality data [32], this platform was chosen for recruitment. By the end of recruitment, advertisements were posted in 59 locations within 47 states and Washington, D.C. 

Individuals interested in participating in the study completed an online screening questionnaire; for this screening questionnaire, a captcha was used to reduce “bots” from completing the questionnaire. Individuals who reported being 18 years or older, using menthol cigarettes for some days or every day during the previous month, and identifying with one or more minoritized sexual orientation and/or gender identity label(s) were eligible to participate. Broad criteria for menthol cigarette use were used to ensure that the results would be generalizable to SGM individuals with any amount of menthol cigarette use. Eligible SGM individuals were invited to participate in the study via the study website (https://groupwisdom.com/, (accessed on 1 November 2021); this site is a social research platform where concept mapping procedures occur) [33]. Participants completed a survey evaluating demographic characteristics, SGM identities, and tobacco use behaviors. Then, participants were asked to complete between one and three concept mapping tasks (i.e., brainstorming, sorting, and rating), depending on which part of the study was open at the time of enrollment. Participants were compensated USD 10, USD 25, and USD 10 via Amazon gift cards for the successful completion of these tasks, respectively. 

### 2.2. Participant Questions Survey

#### 2.2.1. Demographic Characteristics

The demographic questions used in this study are provided in Supplementary Measure S1. All responses were reviewed for consistency with screening questionnaire responses and were excluded if responses did not match. Sexual orientation, gender identity, racial/ethnic identity, income, U.S. region, and health insurance status were included in analyses. Due to small sample sizes, small group sizes for certain identities and characteristics, and limitations of the available analyses, some of the characteristic levels were reduced and combined. The 13 sexual orientation identities were recoded into 5 groups for pattern matching (straight/heterosexual, same-sex/gender attraction, attraction to two or more genders, queer/questioning identities, and asexual/demisexual identities) and 2 groups for *t*-tests (single-gender vs. 2+ gender attraction). The 22 gender identity labels were recoded into 6 groups for pattern matching (cisgender men, cisgender women, transgender men, transgender women, gender-expansive identities, and queer/questioning identities) and 2 groups for *t*-tests (transgender and gender-diverse (TGD) vs. cisgender). Sex assigned at birth was recoded into a dichotomous variable of male and female. Racial/ethnic identity was grouped into four groups for pattern matching (White and European; Black, African, and Caribbean; American Indian, Asian or South Asian, Hispanic, and Latine; and multiracial/ethnic individuals) and two groups for *t*-tests (White vs. non-White). Income skewed toward lower incomes and was split into two categories of USD 0–39,999 and USD 40,000 or more for analysis. Health insurance status was analyzed using two groups (no insurance and public insurance), as no participants indicated that they had private insurance.

#### 2.2.2. Tobacco Use Behaviors

Participants were asked to identify which tobacco products they had used within the previous 30 days and any menthol products other than cigarettes that they were currently using. As a proxy for dependence [34], participants were also asked to report how soon after waking they typically smoked their first menthol cigarette of the day. Participants were additionally asked to report their current level of interest in quitting smoking and whether they were currently attempting to quit smoking.

### 2.3. Concept Mapping Procedures 

#### 2.3.1. Brainstorming 

Participants recruited during the first part of the study were asked to complete the brainstorming task via the groupwisdom website; this task occurred between 1 November 2021 and 2 February 2022. For this task, participants responded to the focus prompt: “If menthol in cigarettes was banned, a specific action I would take related to my tobacco use is…”. Participants were encouraged to provide multiple statements, and all statements were submitted anonymously. A list of statements previously entered by participants was visible to new participants, and new statements were instantly added to this collective list. Participants were instructed to review the list of statements before adding responses to prevent the repetition of statements. This methodology generates more [35] and unique [36] ideas compared to verbal group brainstorming (e.g., focus groups) and prevents interference from having to wait to speak. Because content saturation (i.e., enrolling additional participants no longer yields novel content) guided brainstorming participant enrollment, researchers reviewed statements and closed the brainstorming task when content saturation was reached. A total of 51 participants completed brainstorming, generating a list of 195 statements.

#### 2.3.2. Statement Reduction

Three researchers independently reviewed all statements. After identical duplicate statements were removed, the team identified statements containing multiple ideas and broke these into separate statements. Lastly, the researchers independently identified statements that did not follow the prompt or had redundant content; if two or more researchers identified such a statement, it was removed from the list. For duplicative statements, the statement that best captured the main idea was retained, and the remaining redundant statements were removed. All decisions were made based on group consensus. After review, a final list of 82 statements was retained.

#### 2.3.3. Sorting

Participants who completed the brainstorming task were invited back to the groupwisdom website to complete part two of the study, which included the sorting and rating tasks. This task occurred between 17 February and 24 April 2022. Based on previously established methods [37,38], participants were asked to complete the sorting task by categorizing the statements into piles based on their views of common meanings and themes. Each pile was required to contain a single meaning/theme. The piles were not allowed to be organized based on priority or personal values (e.g., “important”, “would never do”), and “other/miscellaneous” piles were also not allowed. The research team reviewed statement sorting for completion and accuracy according to task instructions. If a participant did not complete the sorting task correctly, the research team sent additional instructions with examples and provided them with another chance to complete the task. 

Of the 51 participants who successfully completed the brainstorming task, 37 (72.5%) returned to complete the sorting and rating tasks; however, only 29 of the original 51 brainstorming task completers (56.9%) successfully completed the sorting task. Thus, additional participants were recruited, which resulted in 47 participants successfully completing the sorting task, overall. This approach to recruiting additional participants is common practice with concept mapping and resulted in a participant sample size for sorting that is consistent with the number of participants needed to optimize the final model fit based on a pooled analysis of concept mapping studies [39]. This was confirmed after reviewing the groupwisdom-calculated stress value (a goodness-of-fit measure) and described further below. 

#### 2.3.4. Rating

Participants were asked to rate each statement on a scale of 0 (definitely not true for me) to 6 (definitely true for me) based on the prompt, “this is an action that I may take if menthol in cigarettes was banned”. This task occurred at the same time as sorting. Responses were reviewed for completion and distinct/repetitive patterns indicative of poor and/or false data, (data from participants with incomplete responses and distinct/repetitive patterns were excluded). Overall, 37 of the 51 brainstorming participants returned to complete the rating task (72.5%). After additional recruitment to account for attrition, a total of 61 participants successfully completed the rating task for this study. According to prior work [39], this sample size was determined to be sufficient for multi-dimensional scaling analyses; thus, data from these 61 participants were used to complete study analyses.

### 2.4. Representation 

Using groupwisdom’s built-in software, an 82 × 82 matrix of similarities was created from participant sorting data, wherein each cell value represented the number of times the two statements had been sorted into the same pile by participants. Non-metric multidimensional scaling (MDS) was used to generate a “point map”, wherein a single point within a two-dimensional space was assigned to each statement. An algorithm for multidimensional scaling [40] determined the location of the points on the map; statements more frequently sorted into the same pile were placed closer to one another on the point map, thus representing content similarity. The stress value of the MDS analysis, an indicator of congruence between the scaled and raw sorting data, was 0.23. This was consistent with previous concept mapping studies [39] and indicated good congruence between raw sorting data and scaled data.

**Figure 1:**
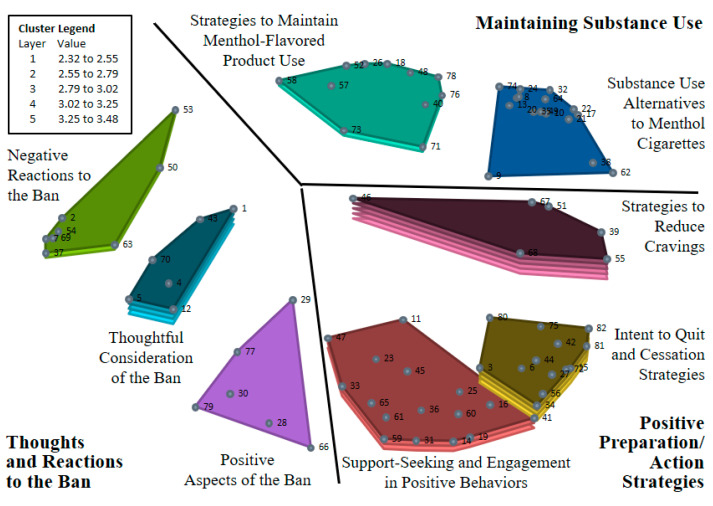
Concept map displaying the 8 clusters of SGM menthol cigarette smoker-identified statements describing reactions to a hypothetical ban on menthol in cigarettes. Numbered points on the map that are closer to one another represent statements of more similar content, whereas points on the map that are further apart represent statements of less similar content. A greater number of layers in the clusters indicates higher mean ratings of statements within the cluster (based on responses to the rating task). Mean ratings for the clusters vary based on the number of layers within that cluster, as indicated by the cluster legend.

Using an algorithm for a hierarchical cluster analysis [41] to identify non-overlapping “clusters” of statements by identifying groups of statements that limited the distance from individual points to the centroid of identified clusters, a hierarchical cluster analysis generated multiple cluster maps. Specifically, the CM software first quantitatively identified a two-cluster model, and subsequent models were built by separating one cluster from the previous model into two clusters using the same methodology of identifying clusters (by limiting the distance between points and the centroid of the cluster). The goal of this hierarchical cluster analysis is to identify the most parsimonious model (i.e., the fewest number of clusters is preferred) in which each cluster only relates to a single theme. Researchers reviewed the cluster models and, through group discussion, examined whether the models met interpretability (i.e., each cluster describing a single theme) and parsimony criteria. The research team determined that the best-fitting model was achieved with eight clusters (see Figure 1). Mean cluster ratings were calculated as the average ratings for all statements within a single cluster. Participant subgroups’ mean cluster ratings were compared using Welch’s *t*-tests for unequal variances and 0.05 significance levels.

## 3. Results

### 3.1. Participant Characteristics

Sample characteristics are displayed in Table 1. Overall, 72 individuals completed participant questions. Participants’ average age was 33.15 (SD = 10.07). The majority of participants reported having at least some college education (79%), an income below USD 60,000 during the previous year (86%), and public insurance (75%). A sizeable minority of participants identified as European or White (43%), followed by Black, African, or Caribbean (26%). Those assigned female at birth (53%) comprised the majority of the sample, and the most commonly reported gender identities—following those of cisgender women (28%) and men (24%)—were gender-expansive (e.g., non-binary, genderqueer, gender fluid; 22%) and queer/questioning (19%) identities. The most commonly reported sexual identities were those indicating attraction to two or more sexes/genders (e.g., bisexual, pansexual; 61%) and those reporting same-gender attraction (i.e., gay, lesbian; 22%). 

### 3.2. Concept Mapping Results

Eight thematic clusters were identified and organized into a cluster map (see Figure 1 and Table 2). These clusters fell under three broad constructs: Thoughts and Reactions to the Ban, Positive Preparation/Action Strategies, and Maintaining Substance Use. 

#### 3.2.1. Thoughts and Reactions to the Ban 

The first group of clusters included statements related to positive, negative, or neutral thoughts or emotional reactions to the ban. The cluster within this group that possessed the highest rating value was the *Thoughtful Considerations of the Ban* cluster (mean cluster rating (M) = 3.13, SD = 0.79, *n* = 6 statements). The statements in this cluster described participants’ interests in learning more about the ban and a level of uncertainty/neutrality toward the ban. The highest-rated statements within this cluster (i.e., those rated as more true for participants) described researching how and why the ban happened, interest in how others felt about the ban, and wanting to know if the majority of people who smoke were supportive of or against the ban. 

The second highest-rated cluster in this group was *Negative Reactions to the Ban* (M = 2.67, SD = 0.57, *n* = 8). The statements in this cluster described negative feelings, stress responses, and intentions to protest the ban. The highest-rated statements in this cluster referred to stress, anger, and gaining weight.

The last cluster in this group was *Positive Aspects of the Ban* (M = 2.35, SD = 0.94, *n* = 6). The statements in this cluster described the potential for positive feelings and outcomes following the ban. The highest-rated statements in this cluster described saving money and feeling reassured that people’s health was of concern and changes were being made.

#### 3.2.2. Positive Preparation/Action Strategies 

The second group of clusters included statements related to intentions and methods of quitting or reducing smoking, including alternative activities, resources, and sources of support. The highest-rated cluster in this group was *Strategies to Reduce Cravings* (M = 3.48, SD = 0.40, *n* = 6). The statements in this cluster described seeking out methods of easing cravings if menthol cigarettes were no longer available. The highest-rated statements in this cluster described seeking other options to satisfy smoking urges and seeking stronger methods of calming nerves. 

The second highest-rated cluster in this group was *Intent to Quit and Cessation Strategies* (M = 3.18, SD = 0.43; *n* = 13). The statements in this cluster tended to describe behaviors that individuals would engage in on their own. Within this cluster, participants shared sentiments related to quitting or reducing smoking, such as trying to reduce nicotine dependence before the implementation of the ban, buying fewer cigarettes following the ban, and trying to stop smoking altogether. Engagement in certain activities, such as cleaning, meditation, and yoga, was also shared. The highest-rated statements in this cluster described seeking out alternative ways to control stress and finding a hobby to distract from smoking.

The final cluster in this group was *Support-Seeking and Engagement in Positive Behaviors* (M = 2.97, SD = 0.49, *n* = 16). This cluster represented one of the two largest clusters in this dataset. The statements in this cluster described places that participants may turn to for support and alternative activities they may engage in to aid in smoking cessation attempts. For instance, participants indicated they would turn to friends, family, therapists, and social media to acquire support. Participants also described alternative ways in which they might invest their time and energy, such as exercising, doing things to benefit the lives of others, and involving themselves in the community. The highest-rated statements within this cluster described going to the Internet, being more involved in their health, and asking friends what they were planning to do. 

#### 3.2.3. Maintaining Substance Use 

The final group was comprised of two clusters that included statements related to methods of maintaining access to menthol-flavored products and substances that may be used as alternatives to menthol cigarettes. The highest-rated cluster in this group was *Strategies to Maintain Menthol-Flavored Product Use* (M = 2.75, SD = 0.58, *n* = 11). The statements in this cluster described methods of subverting the ban to maintain menthol use. The highest-rated statements described buying as many cartons of menthol cigarettes as possible before the ban and stocking up on disposable menthol vapes. 

The final cluster in this group was *Substance Use Alternatives to Menthol Cigarettes* (M = 2.32, SD = 0.87, *n* = 16). This cluster represented the second of the two largest clusters in this dataset. The statements in this cluster described substances that may be used to replace menthol cigarettes. The highest-rated statements in this cluster described vaping menthol and other mint products and trying menthol-flavored nicotine replacement products. Conversely, dip, cigars, and cloves were the lowest-rated alternative products among participants.

### 3.3. Cluster Rating Comparisons

#### 3.3.1 Demographic Characteristics 

In order to perform *t*-test significance testing, demographic characteristics were collapsed to form binary variables. While *t*-tests revealed that mean cluster ratings were not significantly associated with sex assigned at birth, gender identity, or sexual orientation, some clusters were significantly associated with race/ethnicity, income, and health insurance status. Those who identified as White (M = 1.963; t(30) = 2.131, *p* = 0.041) and reported an income of less than USD 40,000 (M = 1.967; t(30) = 2.942, *p* = 0.006) rated statements in the *Substance Use Alternatives to Menthol Cigarettes* cluster significantly lower than those not identifying as White (M = 2.626) and reporting an income of USD 40,000 or more (M = 2.907), respectively. Further, those who reported an income of less than USD 40,000 (M = 2.278; t(30) = 4.686, *p* = 0.000) and those who had public insurance (M = 2.565; t(20) = 2.222, *p* = 0.038) rated statements in the *Strategies to Maintain Menthol-Flavored Product Use* significantly lower than those reporting an income of USD 40,000 or more (M = 3.487) and those without insurance (M = 3.178), respectively. 

#### 3.3.2 Tobacco Use Characteristics

As shown in Table 3, those who reported smoking their first cigarette of the day at least 30 min after waking (*p* < 0.001), those currently interested in quitting smoking (*p* < 0.05), and those currently trying to quit smoking rated statements in the *Negative Reactions to the Ban and Strategies to Maintain Menthol-Flavored Product Use* significantly lower than their counterparts. Conversely, those smoking their first cigarette at least 30 min after waking (*p* < 0.05) and those with a current interest in quitting (*p* < 0.01) rated statements in *the Intent to Quit and Cessation Strategies* significantly higher than their counterparts. Further, statements within the *Intent to Quit and Cessation Strategies* (*p* < 0.001) and *Support-Seeking and Engagement in Positive Behaviors* (*p* < 0.01) were rated significantly higher by those who reported currently using other menthol products and those reporting other tobacco product use during the previous 30 days. 

### 3.4. Pattern Match Comparisons

**Figure 2:**
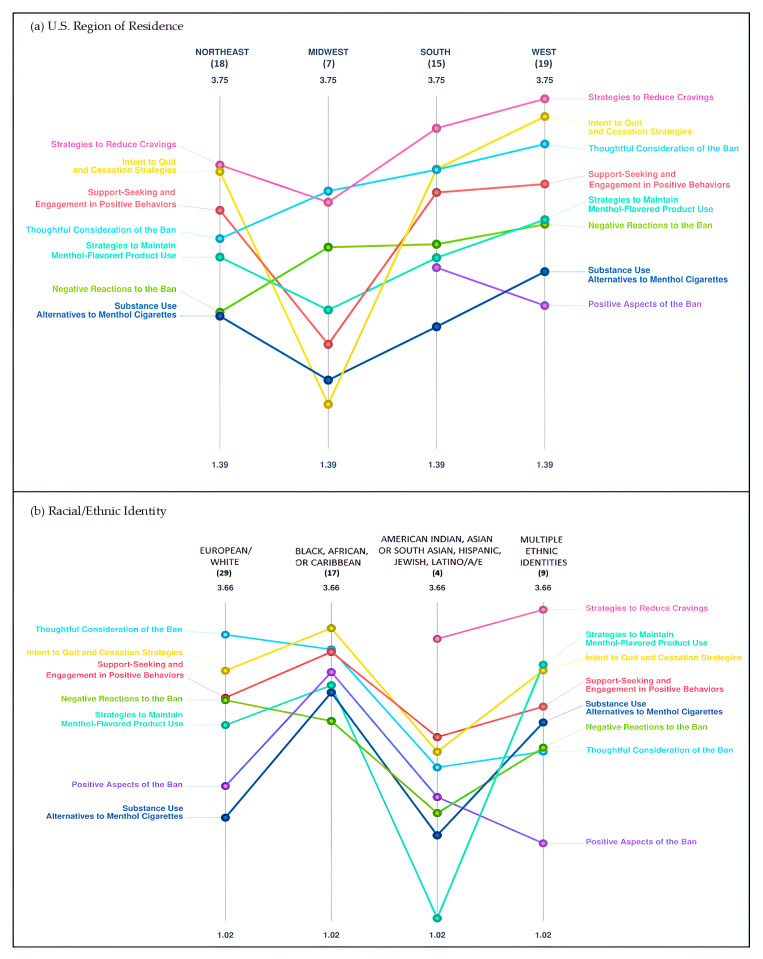
(**a**,**b**) Pattern-matching results comparing cluster rating averages for multi-level demographic variables: (**a**) U.S. region; (**b**) racial/ethnic identity. Numbers in parentheses represent subgroup sample sizes.

**Figure 3:**
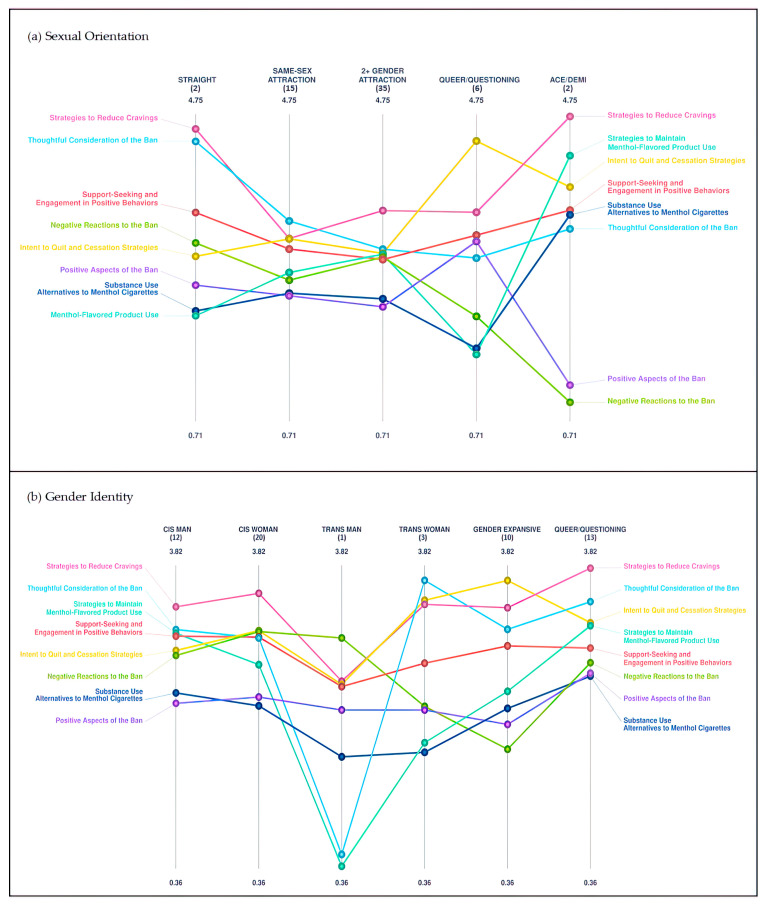
(**a**,**b**) Pattern-matching results comparing cluster rating averages for multi-level SGM variables: (**a**) sexual orientation; (**b**) gender identity. Numbers in parentheses represent subgroup sample sizes.

Mean cluster ratings for each cluster by categorical demographic characteristics were obtained using pattern-matching comparisons and are displayed in Figure 2 and Figure 3. Pattern matches provide a visual representation of cluster rating trends for variables with more than two levels using ladder graphs. Although the pattern match graphs are strictly visual representations of the data and statistical significance cannot be determined, they show that the value placed on the clusters may differ based on individuals’ identities and lived experiences. 

Figure 2 and Figure 3 show pattern-matching graphs for U.S. region, racial/ethnic identity, sexual orientation, and gender identity, and some examples of apparent differences across regions and groups are described below. For example, for U.S. region, participants in the Midwest appeared to place a lower value on items within the *Intent to Quit and Cessation Strategies*, *Support-Seeking and Engagement in Positive Behaviors*, and *Positive Aspects of the Ban* clusters than those residing in other regions. 

For race/ethnicity, participants identifying as Black, African, or Caribbean appeared to rate Positive Aspects of the Ban higher than other groups. Those identifying as American Indian, Asian or South Asian, Hispanic, Jewish, or Latine appeared to rate statements within the *Strategies to Maintain Menthol-Flavored Product Use* cluster lower than all other groups. 

For sexual orientation, those identifying as queer or questioning appeared to differ the most, rating the *Intent to Quit and Cessation Strategies* and *Positive Aspects of the Ban* clusters higher, and the *Negative Reactions to the Ban*, *Strategies to Maintain Menthol-Flavored Product Use*, and *Substance Use Alternatives to Menthol Cigarettes* clusters lower, than all other orientation groups. Those who identified as asexual or demisexual appeared to value the *Strategies to Reduce Cravings*, *Strategies to Maintain Menthol-Flavored Product Use*, and *Substance Use Alternatives to Menthol Cigarettes* clusters more, and the *Positive Aspects of the Ban* and *Negative Reactions to the Ban* clusters less, than the other groups. 

For gender identity, transgender men appeared to value *Strategies to Reduce Cravings*, *Thoughtful Consideration of the Ban*, and *Strategies to Maintain Menthol-Flavored Product Use* clusters lower than all other identity groups. Transgender women appeared to rate the *Thoughtful Consideration of the Ban* cluster higher than all other groups.

## 4. Discussion

It has been suggested by multiple health agencies and community organizations that a menthol ban would have positive public health outcomes for marginalized groups, including SGM communities [4,20]. However, as the FDA pursues a ban on menthol in cigarettes, it is imperative that regulatory officials and public health professionals hear from and consider the voices of the communities most affected by menthol cigarettes. Multiple studies have evaluated how potential regulations may influence product demand and substitution among tobacco users [18,42] and have found that participants’ product consumption matched their product use intentions [43]. 

This study used CM to identify eight thematic clusters of potential behavioral responses to a hypothetical ban on menthol cigarettes, as described by SGM people who smoke menthol cigarettes. Potential behavioral responses included thoughtful and emotional reactions, cessation interest and attempts, and methods of maintaining substance use. Participants were most likely to identify the use of *Strategies to Reduce Cravings* as their response to a menthol cigarette ban; however, ratings for all of the clusters were relatively low. 

In this study, current tobacco use behaviors, including other menthol and tobacco product use, time to first menthol cigarette, and intent and attempts to quit smoking, were associated with greater endorsement of some clusters. Those smoking their first cigarette at least 31 min after waking (indicating lower dependence) and those with an interest in quitting indicated lower ratings for the *Negative Reactions to the Ban* and *Strategies to Maintain Menthol-Flavored Product Use* clusters, as well as higher ratings for the *Intent to Quit and Cessation Strategies* cluster. 

These results may indicate that those with lower nicotine dependence and those interested in quitting smoking would see the ban as a chance to change their smoking behavior. However, these findings are made more interesting when considering that those reporting other menthol or tobacco product use reported higher ratings for *Intent to Quit and Cessation Strategies* and *Support-Seeking and Engagement in Positive Behaviors*. A closer examination of the statements in the *Intent to Quit and Cessation Strategies* cluster reveals ambiguity within some items that may require further study. For example, while some statements in this cluster suggest efforts to pursue smoking cessation and prepping for cessation before the ban is implemented, others might indicate an openness to trying other tobacco products in place of or before cessation (e.g., pursuing alternative methods of stress control, smoking cessation if pleasant alternatives are not available). 

Interestingly, statements within the *Support-Seeking and Engagement in Positive Behaviors* cluster may indicate that those who use other menthol/tobacco products would be open to external support following a menthol ban. While statements regarding seeking support from family were among the lowest-rated within the cluster, turning to the Internet and seeking support from friends were among the highest-rated statements. This finding is in line with other literature describing the tendency for SGM individuals to seek social support outside of their family of origin [44] and for smoking to be related to seeking social connection [45]. Further, this may also point to promising avenues for intervention via messaging campaigns about quitting with friends, online ads for support resources, and online cessation support programs. Others have reported success in supporting cessation attempts using Internet or messaging campaigns [46,47,48], and electronic-based interventions have been shown to be favorable among SGM populations for other substance-related concerns [49]. This work should be expanded upon to determine the acceptability and feasibility of such smoking cessation supports—among SGM individuals, specifically—following a ban on menthol. 

Other highly rated statements related to smoking cessation attempts described exercising and being more involved in improving personal health. This may indicate that some SGM individuals who smoke menthol cigarettes would see the ban as an opportunity to engage in cessation. This insight provides opportunities for intervention and messaging development and should be investigated further before the implementation of a ban on menthol in cigarettes. 

Some of the highest-rated statements across clusters indicate that many SGM individuals who smoke menthol cigarettes would be looking for alternative methods of controlling stress and calming nerves [50,51,52]. This finding supports previous literature on minority stressors unique to SGM communities, such as stigma, prejudice, and discrimination, and their involvement in exacerbating stress [45,53]. Thus, it is likely that SGM adults who currently smoke menthol cigarettes would be in need of additional support to mitigate stress following a menthol ban. These findings further align with the minority strengths model developed by Perrin and colleagues [54], which shows that high levels of resilience and social support promote quicker recovery from stressful situations, improved mental health, and engagement in positive health behaviors. When developing messaging and interventions aimed at mitigating negative responses to a ban on menthol in cigarettes, they should be tailored for SGM communities to improve receptivity [55], and the minority strengths model can provide alternative avenues for stress control.

Support for the ban was low among the present sample, which is in line with some previous work [16] showing a lack of support for policies that restrict the sale of flavored tobacco products among SGM individuals. Low ratings for support of the ban may provide important avenues for messaging before implementation of the ban that explains the public health intentions behind the regulation, details the relationship between nicotine and stress levels, and provides support and resources for effective stress reduction. Overall, regulators may want to focus surveillance efforts on the higher-rated statements/clusters as participants indicated that these reactions may be more likely. 

Finally, although the interpretability of the pattern match graphs is limited, as they are not accompanied by significance testing, they demonstrate how behavioral responses to a menthol ban may vary based on the intersections of identities and life experiences. These intersecting experiences must be considered during the development of messaging campaigns, interventions, and cessation support services. For this reason, and because understanding these intersections allows for better tailoring of each of these initiatives, they are worthy of further study.

### Limitations

The present study has some limitations. The use of Craigslist resulted in an online convenience sample that may underrepresent those with limited or no Internet access. Although the authors are not aware of research comparing Craigslist samples to national samples or the general population, it is possible that our sample differed from these in some meaningful ways. Additionally, the overall sample size was relatively small, as were the sample sizes of some of the SGM identity groups. As a result of this limitation, more nuanced investigations of potential differences in behavioral responses within SGM identity groups could not be completed, and results for the cluster rating comparisons and pattern match should be interpreted with caution. Further, the present sample was not limited to SGM individuals who reported exclusive or predominant menthol cigarette use. Although the present results indicated that more dependent individuals (as defined by time to first menthol cigarette of the day) rated some clusters differently, a sample only comprised of individuals who are daily, dependent menthol smokers might have responded differently. This is an area that should be investigated in future work. 

Despite these limitations, the present study provides community-generated responses to a potential ban on menthol in cigarettes from a national sample of SGM individuals who smoke menthol cigarettes with a diverse range of SGM identities represented. Considering that the disproportionate influence of menthol cigarettes on SGM populations has served as a significant motivation for implementing a ban on menthol in cigarettes, it is imperative that the potential outcomes of such a policy change within this minoritized community continue to be investigated before implementation. 

## 5. Conclusions

Findings from the present study provide community-generated insight into potential behavioral responses that SGM individuals who smoke menthol cigarettes may have following a ban on menthol. The communities that are disproportionately affected by menthol cigarettes are also the most likely to be influenced by a policy change banning menthol in cigarettes; therefore, it is critical that the voices of those from these most-affected communities be considered while developing the policy and before implementation of the ban. The present results can contribute to public health prevention and intervention efforts, messaging campaigns, and support services geared toward SGM individuals who smoke menthol cigarettes, specifically. 

## Figures and Tables

**Table 1:** Sample demographic characteristics.

Characteristic		*Mean*	*(SD)*
		Percentage	(n)
Age, years		*33.15*	*(10.07)*
U.S. Region			
	Northeast	29.2	(21)
	Midwest	15.3	(11)
	South	20.0	(18)
	West	30.6	(22)
Education ^A^			
	9th grade–GED		
	9th, 10th, or 11th grade	2.8	(2)
	12th grade, no diploma	1.4	(1)
	High school graduate, high school diploma	11.1	(8)
	GED (general education diploma)	5.6	(4)
	Some college, no degree		
	Some college credit, but less than 1 year	15.3	(11)
	1 or more years of college, no degree	29.2	(21)
	Associate’s degree+		
	Associate’s degree	11.1	(8)
	Bachelor’s degree	18.1	(13)
	Master’s degree	4.2	(3)
	Professional degree (e.g., MD)	1.4	(1)
Income ^A^			
	USD 0–39,999		
	USD 0–USD 19,999	40.3	(29)
	USD 20,000–USD 39,999	27.8	(20)
	USD 40,000+		
	USD 40,000–USD 59,999	18.1	(13)
	USD 60,000–USD 79,999	9.7	(7)
	USD 80,000–USD 99,999	2.8	(2)
	USD 100,000–USD 119,999	1.4	(1)
	USD 120,000+	0.0	(0)
Insurance Status ^A^			
	No insurance	25.0	(18)
	Public insurance (Tricare, Medicaid, Medicare, veterans benefits)	75.0	(54)
	Private insurance (plans through employers, the Marketplace, parents, and universities, federal employee plans)	0.0	(0)
Racial/Ethnic Identity ^A^			
	American Indian/Asian or South Asian/Hispanic/Jewish/Latino/a/e ^B^	5.6	(4)
	American Indian	1.4	(1)
	Asian or South Asian	2.8	(2)
	Hispanic	2.8	(2)
	Latino/a/e	1.4	(1)
	Black, African, or Caribbean ^B^	26.4	(19)
	African or Caribbean	4.2	(3)
	Black or Brown American	23.6	(17)
	European or White ^B^	43.1	(31)
	Multiethnic ^C^	19.4	(17)
	American Indian	6.9	(5)
	Asian or South Asian	2.8	(2)
	Black or Brown American	5.6	(4)
	European or White	18.1	(13)
	Hispanic	8.3	(6)
	Jewish	6.9	(5)
	Latino/a/e	5.6	(4)
Sex Assigned at Birth ^A^			
	Male ^B^	43.1	31
	Female ^B^	52.8	38
	Intersex	4.2	3
Gender Identity ^A^			
	Cisgender man ^D^	23.6	(17)
	Cisgender man	15.3	(11)
	Man	11.1	(8)
	Cisgender woman ^D^	27.8	(20)
	Cisgender woman	9.8	(7)
	Woman	21.0	(15)
	Transgender man/transmasculine ^D^	2.8	(2)
	Transgender man	2.8	(2)
	Transmasculine	0	(0)
	Man	0	(0)
	Transgender woman/transfeminine ^D^	4.2	(3)
	Transgender woman	4.2	(3)
	Transfeminine	0	(0)
	Woman	0	(0)
	Gender-expansive identities (non-binary, etc.) ^E^	22.2	(16)
	Agender	0	(0)
	Bigender	5.6	(4)
	Demigirl	1.4	(1)
	Genderqueer	1.4	(1)
	Genderflux	1.4	(1)
	Genderfluid	1.4	(1)
	Man	2.8	(2)
	Nonbinary	12.5	(9)
	Pangender	1.4	(1)
	Queer	4.2	(3)
	Questioning	1.4	(1)
	Transfeminine	1.4	(1)
	Transmasculine	1.4	(1)
	Transgender man	2.8	(2)
	Two-spirit	4.2	(3)
	Woman	4.2	(3)
	Queer/questioning ^F^	19.4	(14)
	Cisgender man	1.4	(1)
	Cisgender woman	1.4	(1)
	Queer	18.2	(13)
	Questioning	2.8	(2)
	Man	5.6	(4)
	Transgender man	1.4	(1)
	Transgender woman	1.4	(1)
	Transmasculine	1.4	(1)
	Woman	5.6	(4)
Sexual Orientation ^A^			
	Straight/heterosexual ^B^	4.2	(3)
	Same-gender attraction ^B^	22.2	(16)
	Gay	15.3	(11)
	Lesbian	6.9	(5)
	Same-gender loving	1.4	(1)
	Attraction to 2+ genders ^G^	61.1	(44)
	Asexual	1.4	(1)
	Bisexual	45.8	(33)
	Demisexual	2.8	(2)
	Gay	6.9	(5)
	Homoflexible	2.8	(2)
	Lesbian	6.9	(5)
	Pansexual/polysexual/omnisexual	20.8	(15)
	Queer	15.3	(11)
	Questioning	5.6	(4)
	Same-gender loving	8.3	(6)
	Straight	6.9	(5)
	Queer/questioning ^H^	8.3	(6)
	Gay	2.8	(2)
	Queer	6.9	(5)
	Questioning	1.4	(1)
	Asexual/demisexual ^B^	4.2	(3)
	Asexual	2.8	(2)
	Demisexual	1.4	(1)

N = 72; SD, standard deviation; Variable group percentages add up to 100%, while percentages for identity label options are reported as non-mutually exclusive categories. ^A^ Variables were recoded into discrete categories from multi-selection survey items; variable groupings for analyses are shown with the response options coded into that group shown beneath it. Identity groups were indicated for participants if: ^B^ it was the only identity selected; ^C^ more than one ethnic identity was chosen; ^D^ it was the only identity selected or directly aligned with or opposed participants’ sex assigned at birth; ^E^ participant chose at least one gender-expansive identity, alone or alongside cisgender, transgender, or queer/questioning identities; ^F^ participant chose “queer” or “questioning” identity, alone or alongside non-expansive gender identities; ^G^ multiple-gender attraction identity label was chosen, alone or alongside single-gender attraction; ^H^ participant chose “queer” or “questioning” identity, alone or alongside single-gender attraction.

**Table 2:** SGM menthol cigarette smoker-identified clusters and statements describing reactions to a hypothetical ban on menthol in cigarettes.

Group	Cluster	Statement		Mean Rating
Group 1: Thoughts and Reactions to the Ban				
	Thoughtful Consideration of the Ban			3.13
		1	I would be interested to learn more about menthol cigarettes and how they are more harmful than regular cigarettes.	3.05
		4	I would be interested to know how everyone feels about the ban.	3.62
		5	I would be interested to know if the majority of smokers would be for or against the ban.	3.43
		12	I am not sure of a specific action I would take.	3.25
		43	I would do some research on why and how the ban happened.	3.82
		70	The ban wouldn’t have an effect on me.	1.62
	Negative Reactions to the Ban			2.67
		2	I would lose my mind.	2.26
		7	I would be angry.	3.32
		37	I would be stressed.	3.67
		54	I would cry.	2.12
		69	I would panic.	2.37
		63	I would gain weight.	2.88
		50	I would protest.	2.23
		53	I would begin a petition to have menthol added back into cigarettes.	2.52
	Positive Aspects of the Ban			2.35
		28	I would save money.	3.82
		29	I would get less of my friends accidentally hooked on smoking because they bum a menthol off me and love it.	1.77
		30	I would feel reassured that people’s health is of concern and changes are being made.	2.84
		66	I would be a better thinker.	2.67
		77	I would be in support of the ban.	1.26
		79	I would be happy.	1.74
Group 2: Positive Preparation/Action Strategies				
	Strategies to Reduce Cravings			3.48
		39	I would use tobacco replacement products (e.g., nicotine patches, gum).	2.95
		51	I would look for other options to satisfy my smoking urge.	3.93
		55	I would try using sunflower seeds, hard candy, or chewing gum.	3.05
		67	I would look for a stronger method of calming my nerves.	3.61
		68	I would look for a stronger method of calming my cravings.	3.53
		46	I would eat more.	3.80
	Intent to Quit and Cessation Strategies			3.18
		6	I would seek out alternative ways to control my stress.	4.03
		15	I would try to stop smoking entirely.	3.07
		27	I would quit smoking tobacco.	2.56
		34	I would prepare myself in advance to quit smoking.	3.08
		42	I would take a break from smoking for a week or two and then reassess my habit.	3.02
		44	I would clean.	3.26
		56	I would find a hobby to keep my mind off smoking (e.g., cook, shop, do hair).	3.70
		72	I would do meditation and/or yoga.	2.77
		75	I would try to reduce my nicotine dependence before the ban.	3.41
		81	I would try to quit cold turkey.	2.57
		82	I would stop smoking if I could not find a pleasant alternative.	3.63
		80	I would buy fewer cigarettes.	3.11
		3	I would learn to live without smoking.	3.09
	Support-Seeking and Engagement in Positive Behaviors			2.97
		14	I would exercise more (e.g., walk, jog, run, swim, ride a bike).	3.39
		16	I would stop smoking with friends who are trying to reach the same goal.	3.28
		19	I would start exercising (e.g., walk, jog, run, swim, ride a bike).	3.25
		25	I may ask family for help to stop smoking.	2.60
		31	I would seek consultation from a therapist.	2.67
		33	I would ask my friends what they were planning to do.	3.46
		36	I would study.	2.43
		59	I would be more involved in my health.	3.46
		60	I would do things to involve myself in the community.	2.83
		61	I would do things to better the lives of others.	3.00
		65	I would ask family what would they do or try.	2.44
		45	I would discuss increasing the dosage/amount of my psychiatric treatment with my psychiatrist.	2.20
		23	I would use social media to look for solutions.	3.13
		47	I would go to the Internet.	4.00
		11	I would be less likely to smoke because I can’t smoke non-menthols as much.	2.40
		41	I would get help to stop smoking altogether.	3.00
Group 3: Maintaining Substance Use				
	Strategies to Maintain Menthol-Flavored Product Use			2.75
		18	I would buy them overseas from a different country.	2.60
		26	I would buy menthol cigarettes from other states.	3.18
		40	I would start stocking up on little disposable menthol electronic cigarettes/vapes.	3.36
		48	I would start making my own menthol cigarettes.	2.18
		52	I would buy menthol cigarettes on the street through illegal means.	2.80
		57	I would buy as many cartons of menthol cigarettes as I could afford before the ban started.	3.67
		58	I would sell some menthol cigarettes for profit.	2.20
		71	I would save menthol cigarettes for special occasions.	3.08
		73	I fear I would vape more nicotine than I currently smoke.	3.16
		76	I would research how to make custom smoke blends with herbs.	1.98
		78	I would spend my spare time learning how to cultivate my own tobacco cigarettes.	2.07
	Substance Use Alternatives to Menthol Cigarettes			2.32
		8	I would smoke cloves.	1.35
		10	I would vape other mint products.	3.31
		13	I would start using non-menthol cigarettes.	3.05
		17	I would use dip.	0.90
		20	I would start using cigars.	1.25
		21	I would start using electronic cigars.	2.25
		22	I would use snus or pouches.	1.40
		24	I would increase my cigar use.	1.28
		32	I would use menthol electronic cigarette/vaping products.	3.41
		35	I would start using marijuana.	2.92
		38	I would try menthol-flavored nicotine replacement products.	3.38
		49	I would smoke more marijuana.	2.80
		62	I would try CBD-containing products.	2.93
		64	I would vape electronic cigarettes without menthol flavoring.	2.46
		74	I would purchase herbal smoke blends in bulk.	1.80
		9	I would drink.	2.67

Mean ratings are based on responses to the prompt: “This is an action that I may take if menthol in cigarettes was banned” using a 7-point scale from 0 (Definitely NOT true for me) to 6 (Definitely true for me).

**Table 3:** Welch’s *t*-test results for tobacco use and cessation intention by cluster group and cluster.

	Time to First Menthol Cigarette				Current Interest in Quitting Smoking				Currently Trying to Quit Smoking				Other Menthol Products, Current Use				Other Tobacco Product Use, Past 30 Days		
	0–30 min (*n* = 34) (M)	31+ min (*n* = 25) (M)	Welch’s *T*-Test Results		Yes (*n* = 25) (M)	No/Not Sure (*n* = 33) (M)	Welch’s *T*-Test Results		Yes (*n* = 15) (M)	No/Not Sure (*n* = 44) (M)	Welch’s *T*-Test Results		Yes (*n* = 34) (M)	No (*n* = 25) (M)	Welch’s *T*-Test Results		Yes (*n* = 41) (M)	No (*n* = 18) (M)	Welch’s *T*-Test Results
Group 1: Thoughts and Reactions to the Ban																			
Thoughtful Consideration of the Ban	3.117	3.215	0.213 (*ns*)		2.933	3.350	0.915 (*ns*)		3.144	3.164	0.047 (*ns*)		2.966	3.420	0.925 (*ns*)		3.204	3.056	0.324 (*ns*)
Negative Reactions to the Ban	3.205	1.909	4.362 ***		2.254	2.923	2.409 *		2.103	2.842	2.666 *		2.460	2.933	1.575 (*ns*)		2.613	2.754	0.497 (*ns*)
Positive Aspects of the Ban	2.510	2.100	0.974 (*ns*)		2.554	2.209	0.697 (*ns*)		2.511	2.273	0.492 (*ns*)		2.724	1.796	1.871 (*ns*)		2.500	1.953	0.420 (*ns*)
Group 2: Positive Preparation/Action Strategies																			
Strategies to Reduce Cravings	3.592	3.313	1.186 (*ns*)		3.269	3.643	1.558 (*ns*)		3.389	3.502	0.523 (*ns*)		3.525	3.402	0.472 (*ns*)		3.656	3.056	2.176 (*ns*)
Intent to Quit and Cessation Strategies	3.019	3.423	2.293 *		3.498	2.950	3.092 **		3.430	3.107	1.968 (*ns*)		3.608	2.614	5.754 ***		3.500	2.487	5.789 ***
Support-Seeking and Engagement in Positive Behaviors	3.061	2.844	1.123 (*ns*)		2.980	2.973	0.033 (*ns*)		3.105	2.923	0.829 (*ns*)		3.205	2.645	2.821 **		3.130	2.600	2.761 **
Group 3: Maintaining Substance Use																			
Strategies to Maintain Menthol-Flavored Product Use	2.992	2.321	2.379 *		2.033	3.230	4.689 ***		1.994	2.952	3.870 ***		2.542	2.941	1.475 (*ns*)		2.732	2.660	0.270 (*ns*)
Substance Use Alternatives to Menthol Cigarettes	2.469	2.073	1.212 (*ns*)		1.967	2.579	1.986 (*ns*)		1.925	2.430	1.708 (*ns*)		2.406	2.155	0.805 (*ns*)		2.464	1.926	1.782 (*ns*)

M = mean; * *p* < 0.05; ** *p* < 0.01; *** *p* < 0.001; *ns =* not significant

## Data Availability

The data presented in this study are available upon request from the corresponding author.

## Supplementary Materials

The following supporting information can be downloaded at: https://www.mdpi.com/article/10.3390/ijerph20053891/s1, Supplementary Measure S1: Demographic Questions.

## References

[1] Centers for Disease Control and Prevention (CDC) Current Cigarette Smoking Among Adults in the United States. https://www.cdc.gov/tobacco/data_statistics/fact_sheets/adult_data/cig_smoking/index.htm.

[2] Buchting F.O., Emory K.T., Kim Y., Fagan P., Vera L.E., Emery S. (2017). Transgender Use of Cigarettes, Cigars, and E-Cigarettes in a National Study. Am. J. Prev. Med..

[3] Fallin A., Goodin A.J., King B.A. (2015). Menthol Cigarette Smoking among Lesbian, Gay, Bisexual, and Transgender Adults. Am. J. Prev. Med..

[4] Editorial Staff, American Lung Association The Impact of Menthol Cigarettes on the Health of the LGBTQ+ Community|American Lung Association. https://www.lung.org/blog/menthol-lbgtq-community.

[5] Winickoff J.P., McMillen R.C., Vallone D.M., Pearson J.L., Tanski S.E., Dempsey J.H., Healton C., Klein J.D., Abrams D. (2011). US Attitudes About Banning Menthol in Cigarettes: Results From a Nationally Representative Survey. Am. J. Public Health.

[6] Goodwin R.D., Ganz O., Weinberger A.H., Smith P.H., Wyka K., Delnevo C.D. (2022). Menthol Cigarette Use Among Adults Who Smoke Cigarettes, 2008–2020: Rapid Growth and Widening Inequities in the United States. Nicotine Tob. Res..

[7] Villanti A.C., Collins L.K., Niaura R.S., Gagosian S.Y., Abrams D.B. (2017). Menthol Cigarettes and the Public Health Standard: A Systematic Review. BMC Public Health.

[8] Zeller M. FDA on Track to Take Actions to Address Tobacco-Related Health Disparities 2022. https://www.fda.gov/news-events/fda-voices/fda-track-take-actions-address-tobacco-related-health-disparities.

[9] NIH Sexual & Gender Minority Research Office (SGMRO) (2021). Strategic Plan to Advance Research on the Health and Well-Being of Sexual & Gender Minorities: Fiscal Years 2021–2025.

[10] Pérez-Stable E.J. NIH NIMHD Director’s Message: Sexual and Gender Minorities Formally Designated as a Health Disparity Population for Research Purposes. https://www.nimhd.nih.gov/about/directors-corner/messages/message_10-06-16.html.

[11] U.S. Department of Health and Human Services (USDHHS) LGBT—Healthy People 2030|Health.Gov. https://health.gov/healthypeople/objectives-and-data/browse-objectives/lgbt.

[12] Food and Drug Administration (FDA) (2020). Tobacco Use in the LGBT Community: A Public Health Issue.

[13] U.S. National Cancer Institute (2017). National Cancer Institute. A Socioecological Approach to Addressing Tobacco-Related Health Disparities.

[14] Kowitt S.D., Goldstein A.O., Schmidt A.M., Hall M.G., Brewer N.T. (2017). Attitudes Toward FDA Regulation of Newly Deemed Tobacco Products. Tob. Regul. Sci..

[15] Acosta-Deprez V., Jou J., London M., Ai M., Chu C., Cermak N., Kozlovich S. (2021). Tobacco Control as an LGBTQ+ Issue: Knowledge, Attitudes, and Recommendations from LGBTQ+ Community Leaders. Int. J. Environ. Res. Public. Health.

[16] Acosta-Deprez V., Gorman F.K., Ai M., Chu C., Erlyana E., Records C., London M. (2020). Perceptions About Flavored Tobacco Policies and Smoking Behaviors by Age, Gender and Sexual Orientation in the LGBTQ Population in Los Angeles County. Arch. Healthc..

[17] McGinley L. In a Milestone, FDA Proposes Ban on Menthol Cigarettes, Flavored Cigars. https://www.bostonglobe.com/2022/04/28/nation/milestone-fda-proposes-ban-menthol-cigarettes-flavored-cigars/.

[18] Denlinger-Apte R.L., Cassidy R.N., Carey K.B., Kahler C.W., Bickel W.K., O’Connor R., Thussu S., Tidey J.W. (2021). The Impact of Menthol Flavoring in Combusted Tobacco on Alternative Product Purchasing: A Pilot Study Using the Experimental Tobacco Marketplace. Drug Alcohol. Depend..

[19] Yang Y., Lindblom E.N., Ward K.D., Salloum R.G. (2022). How Smokers of Menthol Cigarettes and Flavored Cigars Might Respond to FDA’s Proposed Bans. Nicotine Tob. Res..

[20] Servigna, Valeria LGBTQ+ Joint Letter to FDA on Menthol 2022. https://cancer-network.org/impact-of-menthol-on-tobacco-disparities-in-the-lgbtq-community/.

[21] Trochim W.M.K. (1989). An Introduction to Concept Mapping for Planning and Evaluation. Eval. Program Plann..

[22] Soule E.K., Nasim A., Rosas S. (2016). Adverse Effects of Electronic Cigarette Use: A Concept Mapping Approach. Nicotine Tob. Res. Off. J. Soc. Res. Nicotine Tob..

[23] Soule E.K., Chaiton M., Zhang B., Hiler M.M., Schwartz R., Cohen J.E., Eissenberg T. (2019). Menthol Cigarette Smoker Reactions to an Implemented Menthol Cigarette Ban. Tob. Regul. Sci..

[24] Soule E.K., Dubray J., Cohen J.E., Schwartz R., Chaiton M. (2021). Smoking Cessation Strategies Used by Former Menthol Cigarette Smokers after a Menthol Ban. Addict. Behav..

[25] Soule E.K., Mayne S., Snipes W., Thomas L., Guy M.C., Breland A., Fagan P. (2022). Electronic Cigarette Users’ Reactions and Responses to a Hypothetical Ban of Flavoured Electronic Cigarette Liquids. Tob. Control.

[26] Miller-Perusse M., Horvath K.J., Chavanduka T., Stephenson R. (2019). Recruitment and Enrollment of a National Sample of Transgender Youth via Social Media: Experiences from Project Moxie. Transgender Health.

[27] Robinson J., Sareen J., Cox B.J., Bolton J. (2009). Self-Medication of Anxiety Disorders with Alcohol and Drugs: Results from a Nationally Representative Sample. J. Anxiety Disord..

[28] Warren J.C., Smalley K.B., Barefoot K.N. (2015). Recruiting Rural and Urban LGBT Populations Online: Differences in Participant Characteristics between Email and Craigslist Approaches. Health Technol..

[29] Alto K.M., McCullough K.M., Levant R.F. (2018). Who Is on Craigslist? A Novel Approach to Participant Recruitment for Masculinities Scholarship. Psychol. Men Masc..

[30] McInroy L.B. (2016). Pitfalls, Potentials, and Ethics of Online Survey Research: LGBTQ and Other Marginalized and Hard-to-Access Youths. Soc. Work Res..

[31] MacDonnell K., Cowen E., Cunningham D.J., Ritterband L., Ingersoll K. (2019). Online Recruitment of a Non-Help-Seeking Sample for an Internet Intervention: Lessons Learned in an Alcohol-Exposed Pregnancy Risk Reduction Study. Internet Interv..

[32] Antoun C., Zhang C., Conrad F.G., Schober M.F. (2016). Comparisons of Online Recruitment Strategies for Convenience Samples: Craigslist, Google AdWords, Facebook, and Amazon Mechanical Turk. Field Methods.

[33] Groupwisdom (2022). GroupwisdomTM—Group Concept Mapping—Social Research 2022.

[34] Muscat J.E., Ahn K., Richie J.P., Stellman S.D. (2011). Nicotine Dependence Phenotype, Time to First Cigarette, and Risk of Head and Neck Cancer. Cancer.

[35] Dennis A.R., Williams M.L. (2003). Electronic Brainstorming: Theory, Research, and Future Directions. Group Creativity: Innovation through Collaboration.

[36] Leggett Dugosh K., Paulus P.B. (2005). Cognitive and Social Comparison Processes in Brainstorming. J. Exp. Soc. Psychol..

[37] Rosenberg S., Park Kim M. (1975). The Method of Sorting as a Data-Gathering Procedure in Multivariate Research. Multivar. Behav. Res..

[38] Weller S., Romney A. (1988). Systematic Data Collection.

[39] Rosas S.R., Kane M. (2012). Quality and Rigor of the Concept Mapping Methodology: A Pooled Study Analysis. Eval. Program Plann..

[40] Kruskal J., Wish M. (1978). Multidimensional Scaling.

[41] Ward J.H. (1963). Hierarchical Grouping to Optimize an Objective Function. J. Am. Stat. Assoc..

[42] Bickel W.K., Pope D.A., Kaplan B.A., DeHart W.B., Koffarnus M.N., Stein J.S. (2018). Electronic Cigarette Substitution in the Experimental Tobacco Marketplace: A Review. Prev. Med..

[43] Quisenberry A.J., Koffarnus M.N., Hatz L.E., Epstein L.H., Bickel W.K. (2016). The Experimental Tobacco Marketplace I: Substitutability as a Function of the Price of Conventional Cigarettes. Nicotine Tob. Res..

[44] Jackson Levin N., Kattari S.K., Piellusch E.K., Watson E. (2020). “We Just Take Care of Each Other”: Navigating ‘Chosen Family’ in the Context of Health, Illness, and the Mutual Provision of Care amongst Queer and Transgender Young Adults. Int. J. Environ. Res. Public. Health.

[45] Antin T.M.J., Hunt G., Sanders E. (2018). The “Here and Now” of Youth: The Meanings of Smoking for Sexual and Gender Minority Youth. Harm. Reduct. J..

[46] Myung S.-K., McDonnell D.D., Kazinets G., Seo H.G., Moskowitz J.M. (2009). Effects of Web- and Computer-Based Smoking Cessation Programs: Meta-Analysis of Randomized Controlled Trials. Arch. Intern. Med..

[47] (2021). Truth Initiative Why We Need More Research on How to Help Young People Quit Vaping. https://truthinitiative.org/research-resources/quitting-smoking-vaping/why-we-need-more-research-how-help-young-people-quit.

[48] Caldwell K.K., Staples A.H., Bnadad L., Lee J.G.L. (2022). Promoting Smoking Cessation Among Lesbian and Bisexual Women: Lessons Learned From a Location-Based Media Campaign in Western North Carolina. Health Promot. Pract..

[49] Meiksin R., Melendez-Torres G.J., Falconer J., Witzel T.C., Weatherburn P., Bonell C. (2021). EHealth Interventions to Address Sexual Health, Substance Use, and Mental Health Among Men Who Have Sex With Men: Systematic Review and Synthesis of Process Evaluations. J. Med. Internet Res..

[50] Bränström R., Pachankis J.E. (2018). Sexual Orientation Disparities in the Co-Occurrence of Substance Use and Psychological Distress: A National Population-Based Study (2008–2015). Soc. Psychiatry Psychiatr. Epidemiol..

[51] Krueger E.A., Fish J.N., Upchurch D.M. (2020). Sexual Orientation Disparities in Substance Use: Investigating Social Stress Mechanisms in a National Sample. Am. J. Prev. Med..

[52] Lehavot K., Simoni J.M. (2011). The Impact of Minority Stress on Mental Health and Substance Use among Sexual Minority Women. J. Consult. Clin. Psychol..

[53] Meyer I.H. (2003). Prejudice, Social Stress, and Mental Health in Lesbian, Gay, and Bisexual Populations: Conceptual Issues and Research Evidence. Psychol. Bull..

[54] Perrin P.B., Sutter M.E., Trujillo M.A., Henry R.S., Pugh M. (2020). The Minority Strengths Model: Development and Initial Path Analytic Validation in Racially/Ethnically Diverse LGBTQ Individuals. J. Clin. Psychol..

[55] Berger I., Mooney-Somers J. (2017). Smoking Cessation Programs for Lesbian, Gay, Bisexual, Transgender, and Intersex People: A Content-Based Systematic Review. Nicotine Tob. Res..

