# Supplementary material for: Reactions to a Hypothetical Menthol Cigarette Ban among Sexual- and Gender-Minoritized Communities: A Concept Mapping Study
Source: Int J Environ Res Public Health. 2023 Feb 22;20(5):3891. doi: 10.3390/ijerph20053891 (PMC10001923; doi:10.3390/ijerph20053891)
Supplement: Supplementary file 1 [file ijerph-20-03891-s001.zip › ijerph-2168702-supplementary.pdf]

Supplementary Measure A1: Demographic Questions.

The following are the demographic questions employed in the study survey. Note that U.S. region was determined from participants' reporting of their state of residence.

Sexual Orientation

Your sexual orientation is: *[please select all that apply]*

1. Asexual
2. Bisexual/ Bi
3. Demisexual
4. Down-low
5. Gay/ homosexual
6. Homoflexible
7. Lesbian
8. Pansexual/ Polysexual/ Omnisexual
9. Queer
10. Questioning
11. Same gender loving
12. Straight/ heterosexual
13. A different orientation (please share your identity**:** ___________)

Gender Identity

Your gender identity is: *[please select all that apply]*

1. Agender
2. Bigender
3. Cisgender man
4. Cisgender woman
5. Demiboy
6. Demigirl
7. Genderqueer
8. Genderflux
9. Genderfluid
10. Man
11. Neutrois
12. Non-binary
13. Pangender
14. Queer
15. Questioning
16. Transgender man
17. Transgender woman
18. Transmasculine
19. Transfeminine
20. Two-spirit
21. Woman
22. A different identity (please share your identity**:** ___________)

Ethnic Identity

What ethnic group(s) do you consider yourself to be? *[please select all that apply]*

1. African or Caribbean
2. Alaskan Native
3. American Indian
4. Asian or South Asian
5. Black or Brown American
6. European or White
7. Hispanic
8. Jewish
9. Latino/a/e
10. Middle Eastern
11. Native Hawaiian
12. Pacific Islander
13. Other (what other ethnic group(s) do you identify with? ______________)

Income

What is your best estimate of your total household income from all sources last year, before taxes?

1. $0 – $19,999
2. $20,000 – $39,999
3. $40,000 – $59,999
4. $60,000 – $79,999
5. $80,000 – $99,999
6. $100,000 – $119,999
7. $120,000 – $139,999
8. $140,000 – $159,999
9. $160,000 – $179,999
10. $180,000 – $199,999
11. $200,000+

Health Insurance

Do you have health insurance?

0. 0, No
1. Yes, public insurance (includes: Military insurance (Tricare), Medicaid, Medicare, veterans benefits)
2. Yes, private insurance (includes: Plans through employers, the Marketplace, parents, and universities; federal employee plans)
3. I’m not sure
